# When a vesicular placenta meets a live fetus: case report of twin pregnancy with a partial hydatidiform mole

**DOI:** 10.1186/s12884-021-04160-2

**Published:** 2021-10-13

**Authors:** Minhuan Lin, Jinzhu Chen, Bing Liao, Zhiming He, Shaobin Lin, Yanmin Luo

**Affiliations:** 1 Department of Obstetrics & Gynecology, the First Affiliated Hospital of Sun Yat-Sen University, No.58, Zhong Shan Er Lu, Guangzhou, China; 2 Department of Obstetrics & Gynecology, Quanzhou First Hospital Affiliated to Fujian Medical University, No.215, Wen Ling Nan Lu, Quanzhou, China; 3 Department of Pathology, the First Affiliated Hospital of Sun Yat-Sen University, No.58, Zhong Shan Er Lu, Guangzhou, China

**Keywords:** Twin pregnancy, Hydatidiform mole, Placental mesenchymal dysplasia, Mosaicism, Case report

## Abstract

**Background:**

Hydatidiform moles exhibit a distinctive gross appearance of multiple vesicles in the placenta. The advances in cytogenetic technologies have helped uncover novel entities of hydatidiform moles and enabled elaborate diagnoses. However, management of a vesicular placenta with a coexistent live fetus poses a bigger challenge beyond hydatidiform moles.

**Case presentation:**

A 33-year-old woman was referred to our department for suspected hydatidiform mole coexistent with a live fetus at 24 weeks’ gestation. The patient had conceived through double embryo transplantation, and first-trimester ultrasonography displayed a single sac. Mid-trimester imaging findings of normal placenta parenchyma admixed with multiple vesicles and a single amniotic cavity with a fetus led to suspicion of a singleton partial molar pregnancy. After confirmation of a normal diploid by amniocentesis and close surveillance, the patient delivered a healthy neonate. Preliminary microscopic examination of the placenta failed to clarify the diagnosis until fluorescence in situ hybridization showed a majority of XXY sex chromosomes. The patient developed suspected choriocarcinoma and achieved remission for 5 months after chemotherapy, but relapsed with suspected intermediate trophoblastic tumor.

**Conclusion:**

We report a rare case of twin pregnancy comprising a partial mole and a normal fetus that resembled a singleton partial molar pregnancy. Individualized care is important in conditions where a vesicular placenta coexists with a fetus. We strongly recommend ancillary examinations in addition to traditional morphologic assessment in such cases.

## Background

Due to the distinctive gross appearance, hydatidiform moles (molar pregnancies) have been described since the fourth century BC [1]. The incidence of hydatidiform moles varies in different ethnic groups, with an estimated incidence of 2.43–13 per 1000 pregnancies in Asians and 0.5–1.84 in Caucasians [2, 3]. Since the late 1970s, hydatidiform moles have been classified as complete and partial hydatidiform moles based on the genetic and histopathologic features [4]. Classically, complete moles are androgenetic diploid, and partial moles are diandric monogynic triploid. The ratio of partial to complete moles is approximately 1.5–1.8 [5, 6]. In recent years, familial biparental complete moles, mosaic complete moles, and tetraploid partial moles have been reported [7]. In 1991, placental mesenchymal dysplasia (PMD) was proposed as a novel pathological entity resembling hydatidiform moles [8]. The estimated incidence of PMD was reported to be two per 10,000 pregnancies, but this is probably an underestimate [9]. Features of PMD are analogous to those of partial moles and may include cystic placenta, elevated human chorionic gonadotropin (HCG) levels, fetal abnormalities, and preeclampsia [10].

Coexistence of a vesicular placenta with a fetus is a rarer phenomenon which encompasses four categories: dizygotic twin pregnancy involving a fetus and a coexistent complete or partial mole; singleton pregnancy of a partial mole with a malformed triploid fetus; a mosaic complete or partial mole with a mosaic fetus, or a mosaic mole with an euploid fetus (confined placental mosaicism, CPM); and PMD [10–17]. Intriguingly, the reported incidence of twin pregnancy with a complete mole is one per 10,000–100,000 pregnancies [18, 19], whilst twin pregnancies with a partial mole are even rare [14], which is possibly due to the high first-trimester miscarriage rate for partial moles [20]. A mosaic complete or partial mole with a fetus is the rarest entity, as reported in a few articles [12–16]. Apart from a possibly malformed fetus, the other potential complications of hydatidiform mole coexisting with a fetus include vaginal bleeding, hyperthyroidism, preeclampsia, fetal death, fetal growth restriction (FGR), preterm birth, hyperemesis, fetal-maternal hemorrhage, and gestational trophoblastic neoplasia (GTN) [13, 14, 21]. The above four categories of coexisting vesicular placenta with fetus have similar presentations but different prognosis. Therefore, prenatal diagnosis and management in these cases is not straightforward owing to the possibility of a healthy fetus.

We report a case of suspected singleton partial mole with a structurally and karyotypically normal fetus. Postnatal placental morphological examination failed to provide a precise diagnosis in the first place. Thus, we investigated the case and performed a literature review of the diagnosis and management of vesicular placenta with a fetus.

## Case presentation

A 33-year-old woman, gravida 2 para 0, was referred to our fetal medicine department for a suspected hydatidiform mole coexistent with a live fetus at 24 weeks’ gestation. Her first pregnancy was a miscarriage around 5 weeks’ gestation conceived by in vitro fertilization (IVF) a year ago. She had no family history of twin pregnancy or hydatidiform mole. She had conceived through transfer of 2 day-5 embryos following IVF although the indication for double rather than single embryo transfer was obscure.

**Fig. 1:**
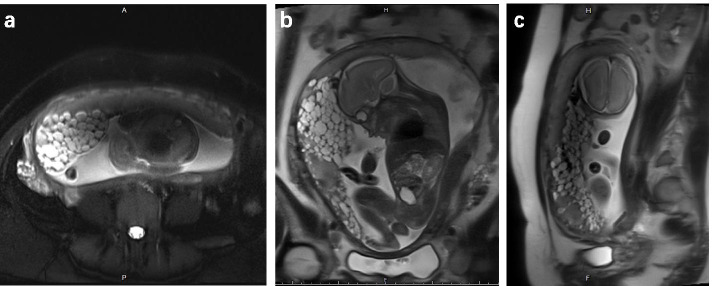
MR images displaying a placenta with a normally-appearing portion and an abutting multicystic mass with high signal on T2-weighted images at the right anterior wall. The mass is partly admixed with the normal placental parenchyma and partly bulged between the normal placental parenchyma and the amniotic sac

Several ultrasound examinations performed during the first trimester displayed a single sac containing a fetus with no signs of placental abnormalities. Her thyroid hormone levels were normal, but her first trimester hCG and prenatal genetic screening results were not available. At the time of her visit, the gestational age was 24 weeks. She had no vaginal spotting and her blood pressure was normal. Her serum β-human chorionic gonadotropin (β-hCG) level was 105,851 IU/L. Ultrasound evaluation revealed a structurally normal fetus with an intraplacental multicystic mass measuring 154 mm × 53 mm; no theca lutein cysts were observed. Magnetic resonance imaging (MRI) showed a placenta with a normally-appearing portion abutted by multicystic mass with high signal on T2-weighted images at the right anterior wall (Fig. 1). The 151 mm × 139 mm × 67 mm multicystic mass was partly admixed with the normal placenta and partly bulged between the normal placenta and the amniotic sac. There were no signs of aneurismal or varicose dilation of fetal chorionic vessels. Her chest X-ray was normal.

Amniocentesis revealed a 46, XX karyotype without pathological copy number variants. At this point the working diagnosis was twin pregnancy with a partial mole or CPM with a partial mole, coexisting with a normal fetus. The patient was counselled regarding the potential maternal and fetal risks associated with pregnancies with hydatidiform moles, following which the patient decided to continue the pregnancy. She was monitored closely. Serial ultrasonographic evaluation of the conceptus performed once every 2 or 3 weeks showed a normally-growing fetus and a persistent and slightly-diminishing multicystic mass measuring 110 mm × 106 mm × 24 mm at 37 weeks’ gestation.

**Fig. 2:**
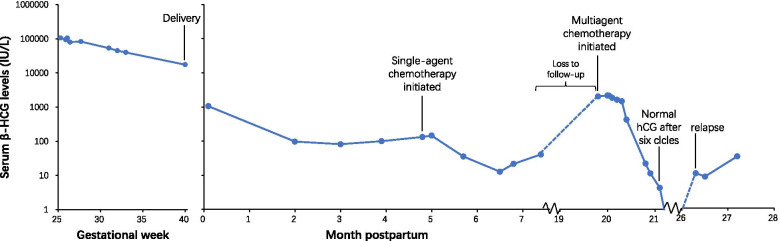
Serum β-HCG level during pregnancy and postpartum follow-up

**Fig. 3:**
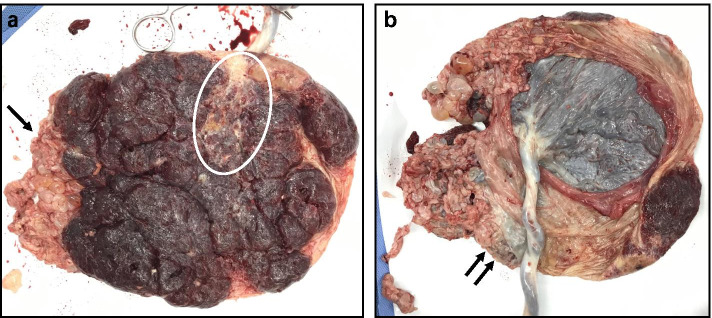
Macroscopic examination of the placenta showing 10 cm × 7 cm × 1 cm multiple edematous cysts of various sizes adhering to a lateral side (black arrow) and fetus side (double black arrow) of the normal-looking placenta and an adjacent part of thin chorionic plate (white circle)

She remained asymptomatic with normal blood pressure. Her serum β-hCG levels peaked around 105,000 IU/L at 25–26 weeks’ gestation and gradually declined to 17,483 IU/L at delivery (Fig. 2). At 40^2/7^ weeks’ gestation, ultrasound examination showed a fetus with an estimated weight of 2745 g (5.4th percentile for the gestational age according to the NICHD fetal growth curve for Asians [22]) and a deepest vertical amniotic fluid pocket of 11 mm. Considering the oligohydramnios and the risk of potential metastasis of trophoblastic cells by uterine contractions during vaginal delivery, cesarean section was performed and a female neonate was delivered without any complications. The Apgar scores at 1 and 5 min were 9 and 10, respectively. The neonate weighed 2840 g (8th percentile for gestational age) and had no gross anomalies. Macroscopic examination of the placenta showed an adjacent mass (10 cm × 7 cm × 1 cm) of multiple cysts of varying sizes (Fig. 3). The mass was partly intermediate between the normal-looking placental parenchyma and chorionic membranes. Thin parenchyma was also noted on the area of chorionic plate near the mass.

**Fig. 4:**
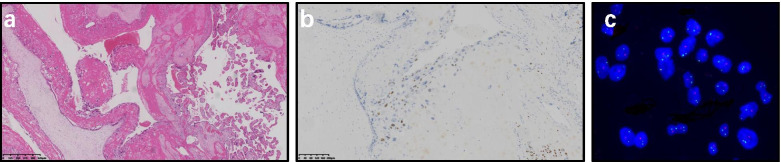
**a** Histopathological images showing large, hydropic villi, cistern formation and circumferential trophoblastic hyperplasia of the cystic mass, adjacent to normal-sized villi of the placentas (hematoxylin-eosin 40×). **b** Focally positive p57^KIP2^ immunostaining of the mole (100×). **c** FISH for the X and Y chromosomes on the slide of molar tissue displaying mostly XXY

Histopathological examination of the normal part of the main placenta revealed features of a normal mature placenta. The cystic mass showed large, hydropic villi, circumferential trophoblastic hyperplasia (Fig. 4a), which was partly necrotic. Histopathological classification (complete or partial mole) was inconclusive. DNA was extracted from a few rinsed macroscopic vesicles, and samples of normal placenta, amniotic fluid, and parental peripheral blood. Twenty short tandem repeat (STR) loci were tested using AmpFlSTR-Identifiler-PCR kit according to the manufacturer’s instructions (Thermo Fisher Scientific). The amniotic fluid and normal placenta DNA showed the same balanced biallelic profiles of both paternal and maternal contributions, with no additional suspicious peaks. However, the vesicular tissue exhibited predominantly all maternal alleles at all loci with pretty small peaks of homozygous or heterozygous paternal alleles at a few loci, which was probably attributable to the contamination of maternal decidua and necrosis of molar tissue.

To rule out familial recurrent complete mole owing to the patient’s history of an early miscarriage and a molar pregnancy, Sanger sequencing of *NLRP7* (19q13.42) and *KHDC3L* (6q13) were performed on genomic DNA extracted from the patient’s blood, which showed no mutations. Immunohistochemical staining for p57^KIP2^ expression in the molar tissue showed focal positivity in the villi (Fig. 4b). Finally, fluorescence in situ hybridization (FISH) for the X and Y chromosomes on the slide of molar tissue was performed. Two hundred cells were scored and XXY was observed in 88% of cells (Fig. 4c). The rest were XXXYY (7%) and XYY (5%), which was considered interference by syncytiotrophoblast and loss of nuclear components due to slicing. Until this point, a partial mole was ascertained despite lack of genetic test results and we favored the diagnosis of a twin pregnancy over CPM due to the relatively clear margin of the molar lesion from the normal placenta and normal chromosomes of the fetus.

The patient’s serum β-hCG was maintained around 100 IU/L 3 months postpartum; thus, she was diagnosed with GTN and underwent four courses of single-agent chemotherapy using dactinomycin. However, she did not follow medical advice to receive the subsequent courses. One year later, chest computed tomography showed several lung nodules suggestive of pulmonary metastases. Core needle biopsy of lung nodules revealed a large area of coagulative necrosis and a small cluster of tumor cells with marked atypia. Immunostaining for CK and beta-hCG were positive and 50% of tumor cells stained positive for p53. The initial diagnosis was reported as metastatic trophoblastic tumor. However, due to the small amount of tumor cells, we were not able to further classify or perform genotyping of lung biopsy.

The patient was diagnosed as choriocarcinoma and received multiagent regimens of EMA/CO (etoposide, methotrexate, actinomycin D, cyclophosphamide and vincristine [oncovin]). Her β-hCG levels returned to normal after six treatment cycles. She received another three cycles and was in remission for 5 months; however, she relapsed showing mild elevation of β-hCG level and increase in lung nodules but no uterine mass was observed. The attending gynecologist suspected intermediate trophoblastic tumor due to the relatively low levels of β-hCG and her insensitivity to chemotherapy. She was scheduled to undergo partial pulmonary lobectomy. Her child is developing normally at 29 months.

## Discussion and conclusions

We report a rare case of twin pregnancy with a partial mole and a coexisting normal fetus. Comprehensive evaluation and close monitoring of the pregnancy led to delivery of a healthy baby. The biggest challenge of this case was the diagnosis. During the gestational period, we were uncertain whether it was a twin pregnancy consisting of a hydatidiform mole and a fetus or a singleton pregnancy of CPM associated with a mole. Prenatal imaging showed intermixing of the multicystic mass with the normal placenta, leading to suspicion of a singleton partial mole. On macroscopic examination of specimen, the mass exhibited a morphology that was partly intermediate between the normal placental parenchyma and chorionic membranes, which was consistent with the prenatal images. However, on gross examination, the molar mass was found to have abutted into the normal placenta (rather than intermixed) and there was a clear boundary between the two elements. In a recent review the percentage of mosaicism in CPM placenta biopsies was 32.5–100% (IQR), with a median of 100% [23]. Thus, the fact that STR of normal placenta DNA showed balanced biparental profiles favored the diagnosis of a twin pregnancy rather than CPM [24]. Early ultrasonography showed a single sac probably because a subtle molar mass is hard to discern by ultrasound if it accompanies a well-developed placenta [21]. Prenatal images with an intermixing pattern could be explained by reduction of tumor size in prenatal imaging and corresponding decrease in serum β-hCG. A focal area of thin chorionic plate in the normal placenta adjacent to the mass possibly represented recession of the mole. Previous reports of twin pregnancies with a hydatidiform mole stated a clear boundary between normal placenta and molar tissue [25]. Such a growing pattern of a hydatidiform mole into another normal placenta has not been described before.

Based on a review of literature, we summarized all conditions wherein a vesicular placenta may coexist with a fetus. These include: (1) dizygotic twin pregnancy with a fetus and a coexistent complete or partial mole; (2) singleton pregnancy of a partial mole with a malformed triploid fetus; (3) a mosaic complete or partial mole with a mosaic fetus, or a mosaic mole with an euploid fetus (CPM); (4) PMD [10–17]. Genetically, karyotypes of complete moles are 46, XX (90%) or 46, XY (10%) [26]; karyotypes of partial moles are 69, XXY, 69, XXX, or 69, XYY (70, 27, 3%) [3]. A mosaic complete or partial mole with a mosaic fetus, or a mosaic mole with a euploid fetus (CPM) is the rarest type which can be confirmed by molecular genotyping [14–16]. PMD is sometimes associated with Beckwith-Wiedemann syndrome (BWS) and androgenetic/biparental mosaicism [27].

In literature review, we identified a few common misconceptions about hydatidiform moles. First, diploid partial moles probably do not exist due to the lack of underlying genetic pathogenesis, but were reported in some case reports [3, 15]. Second, evidence of a fetus in a partial mole could be either an amniotic sac containing a fetus or nucleated red blood cells in villous capillaries - the latter phenomenon is less known [28, 29]. Last but not least, although histologic differentiation of hydropic villi has been described numerously, quite a few studies have demonstrated significant interobserver and intra-observer variability in the diagnosis of hydatidiform moles, hydropic abortion, and PMD based solely on traditional hematoxylin-eosin-stained morphologic assessment, even by specialized pathological experts [30–33]. For example, Malgorzata G et al. reported termination of an initially-diagnosed twin pregnancy with a complete mole and a coexistent fetus based on histopathological assessment; however, they made a final diagnosis of twin with a partial mole by p57^KIP2^ immunostaining reassessment [17]. Therefore, pathological diagnosis may not be the gold standard in molar cases [34].

Researchers have advocated selective use of ancillary techniques such as p57^KIP2^ immunostaining, FISH, STR genotyping (microsatellite genotyping) or SNP-based microarray analysis to refine the diagnosis [6, 30, 35, 36]. P57^KIP2^ is a protein encoded by the imprinted maternally expressed gene *CDKN1C* located on chromosome 11p15.5; thus p57^KIP2^ immunostaining is negative in androgenetic complete moles and diandric triploid partial moles with loss of maternal chromosome 11 [37], while it is positive in different groups of certain cells under other circumstances (biparental complete moles, mosaic complete moles, complete moles with a retaining maternal chromosome 11 [38], partial moles, PMD, non-molar products of conception) [3]. Focally-positive p57^KIP2^ immunostaining observed in our case can be interpreted as positive, as has been clarified in previous studies [30]. FISH with a set of chromosome enumeration probes (CEP) for chromosomes X, Y and other autosomes is another efficient means for arriving at a definitive diagnosis, or literally a critical one as demonstrated in our case.

Molecular genotyping determines the parental source and ratios of polymorphic alleles by comparing villous and parental DNA patterns and is suggested as an ultimate method for diagnosis [30]. In the present case, STR results of molar tissue displayed predominantly two maternal alleles, which we reckon was attributable to contamination by maternal decidua and necrosis of tumor cells. Such contamination has been reported in previous placenta research studies and is sometimes inevitable [38, 39]. This can be avoided if DNA is extracted from serial sections with verification of villous in one hematoxylin-eosin-stained section [40]. In addition, washing the specimen with sterile saline rather than any other liquid better preserves the DNA contents. There is still a shortage of ancillary examinations, especially genetic testing, in cases with a vesicular placenta [15, 35, 41–44]. Thus, ancillary examinations should be recommended for a refined diagnosis.

**Fig. 5:**
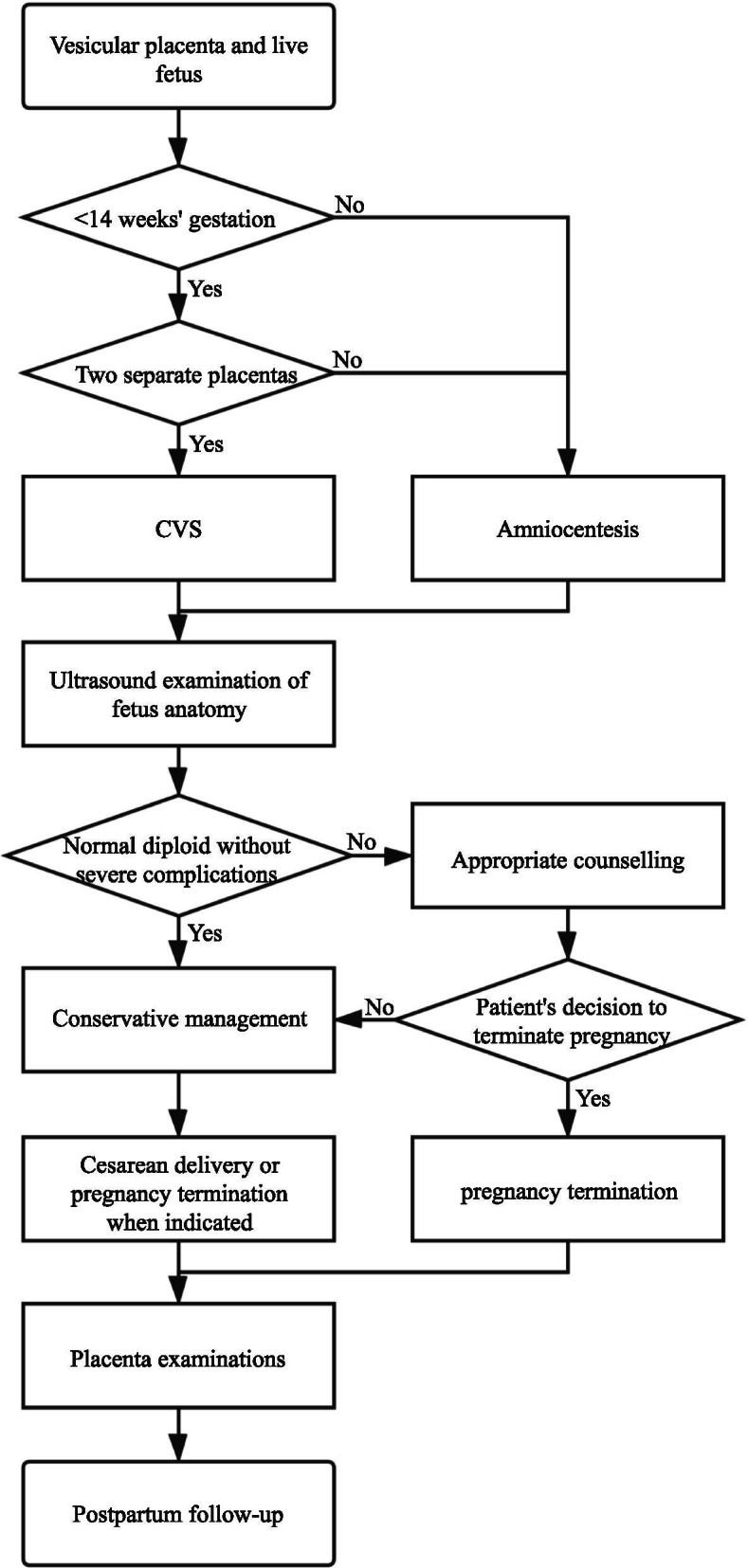
Proposed scheme for management of patients pregnant with a live fetus and a vesicular placenta. CVS: Chorionic villus sampling

If prenatal ultrasound detects a vesicular placenta coexisting with a fetus, patients should be referred to a fetal medicine unit. In this setting, patient evaluation should cover basic checkups, especially maternal serum HCG and alpha-fetoprotein (AFP) levels, thyroid hormones, blood pressure, and ultrasound. Meticulous ultrasound evaluation of fetal anatomical structures, placenta, and adnexa should be performed. If the molar placenta is clearly separated from the normal placenta, a diagnosis of twin pregnancy can be made (proposed scheme shown in Fig. 5); if there is one vesicular placenta, there is low specificity of ultrasound diagnosis, as was observed in the present case. In addition, an invasive prenatal diagnosis (metaphase chromosome analysis and chromosomal microarray analysis) should be performed to confirm the fetal karyotype. Maternal cell-free DNA screening is an additional investigation to analyze placental genetic components or a potential alternative to invasive prenatal testing [45].

Even with normal fetal testing results, patients should be fully informed of the pros and cons of continuing the pregnancy. According to a recent meta-analysis of twin pregnancies with a complete mole and coexistent normal fetus, there are 50% chances of a live birth if the pregnancy is continued; the incidence of complications is 70.5% for vaginal bleeding, 40.1% for intrauterine fetal death, 39% for preterm births, 34% for GTN, 23.3% for hyperthyroidism and 14.3% for preeclampsia [21]. Other possible complications of hydatidiform moles are fetal growth restriction, hyperemesis, theca lutein cyst, and fetal-maternal hemorrhage [12, 20]. The risk of GTN does not increase with advancing gestational age [46], but opinions vary regarding the risk of GTN following a twin pregnancy with a hydatidiform mole versus that after a singleton complete mole. Some have reported similar risk while others have reported a higher risk for the former [20, 47]. Mosaic complete or partial moles probably share these complications with common hydatidiform moles. In a systematic review of cases of PMD, preterm delivery, FGR, and intrauterine fetal death affected 52, 33 and 13% of PMD patients [27].

In the absence of any severe complication necessitating termination of pregnancy and if the patient opts for expectant management, close prenatal monitoring for potential complications and serial ultrasound examinations should be carried out. If there are severe complications or patient chooses to terminate, the methods for pregnancy termination depend on the gestational age. Suction curettage with ultrasound guidance is recommended in early pregnancy. Plans for second-trimester termination should be made according to the patient’s condition and local guidelines. There is no clear consensus on the risks of GTN following different abortion or delivery methods; however, some argue against medical abortion and vaginal delivery due to potential risk of metastasis of trophoblastic cells by uterine contractions [7, 41, 42, 48]. Thus, cesarean section is recommended to deliver a viable fetus. For patients with a hydatidiform mole, regular follow-up should be followed as per guidelines [7, 49].

A strength of our report is that this is the first recorded case of twin pregnancy with a partial mole, with the two placentas displaying an intermixed pattern. We find that there is still room for refined diagnosis in obstetrical molar cases. A limitation of our report is that we could not obtain a satisfactory genotyping result of the molar tissue. Given the limited evidence in our case and the literature, further studies are required to focus on the diagnosis and molecular genotyping in cases of vesicular placenta coexisting with a fetus, genetic basis of PMD, the risk of GTN following a twin pregnancy with hydatidiform mole compared with the risk following a singleton mole, and the risk of GTN following different modes of pregnancy termination or delivery.

In conclusion, this report describes a rare case of a twin pregnancy comprising a partial mole that presented as a singleton mole in prenatal imaging. Coexistence of a vesicular placenta with a fetus is a complex scenario which is tricky to manage. A comprehensive evaluation of maternal-fetal state, including prenatal imaging, fetal genetic testing, and maternal complication screening is required to individualize the management. Selective use of p57^KIP2^ immunohistochemical staining, FISH, or DNA genotyping is strongly recommended to refine diagnosis in addition to placental routine microscopic evaluation.

## Data Availability

The datasets of the current study are not publicly available due to protection of the patient’s privacy but are available from the corresponding author on reasonable request.

## Abbreviations

PMD: Placental mesenchymal dysplasia
hCG: Human chorionic gonadotropin
CPM: Confined placental mosaicism
FGR: Fetal growth restriction
GTN: Gestational trophoblastic neoplasia
IVF: In vitro fertilization
MRI: Magnetic resonance imaging
STR: Short tandem repeat
FISH: Fluorescence in situ hybridization
EMA/CO: Etoposide, methotrexate, actinomycin D, cyclophosphamide, vincristine [oncovin]
SGA: Small for gestational age
CEP: Chromosome enumeration probes
AFP: Alpha-fetoprotein

## References

[1] Richardson MV, Hertig AT (1959). New England's first recorded hydatidiform mole; a historical note. N Engl J Med.

[2] Jacobs PA, Hunt PA, Matsuura JS, Wilson CC, Szulman AE (1982). Complete and partial hydatidiform mole in Hawaii: cytogenetics, morphology and epidemiology. Br J Obstet Gynaecol.

[3] Hui P, Buza N, Murphy KM, Ronnett BM (2017). Hydatidiform moles: genetic basis and precision diagnosis. Annu Rev Pathol.

[4] Lawler SD, Pickthall VJ, Fisher RA, Povey S, Evans MW, Szulman AE (1979). Genetic studies of complete and partial hydatidiform moles. Lancet..

[5] Tham BW, Everard JE, Tidy JA, Drew D, Hancock BW (2003). Gestational trophoblastic disease in the Asian population of northern England and North Wales. BJOG..

[6] Colgan TJ, Chang MC, Nanji S, Kolomietz E (2016). A reappraisal of the incidence of placental Hydatidiform mole using selective molecular genotyping. Int J Gynecol Cancer.

[7] Tidy J, Seckl M, Hancock BW, on behalf of the Royal College of Obstetricians and Gynaecologists. Management of Gestational Trophoblastic Disease: green-top guideline no. 38 - June 2020. BJOG. 2021;128(3):e1–e27. 10.1111/1471-0528.16266 32996207

[8] Moscoso G, Jauniaux E, Hustin J (1991). Placental vascular anomaly with diffuse mesenchymal stem villous hyperplasia. A new clinico-pathological entity?. Pathol Res Pract.

[9] Guenot C, Kingdom J, De Rham M, Osterheld M, Keating S, Vial Y (2019). Placental mesenchymal dysplasia: an underdiagnosed placental pathology with various clinical outcomes. Eur J Obstet Gynecol Reprod Biol.

[10] Ohira S, Ookubo N, Tanaka K, Takatsu A, Kobara H, Kikuchi N (2013). Placental mesenchymal dysplasia: chronological observation of placental images during gestation and review of the literature. Gynecol Obstet Investig.

[11] McNamara HC, Kane SC, Craig JM, Short RV, Umstad MP (2016). A review of the mechanisms and evidence for typical and atypical twinning. Am J Obstet Gynecol.

[12] Crooij MJ, Van der Harten JJ, Puyenbroek JI, Van Geijn HP, Arts NF (1985). A partial hydatidiform mole, dispersed throughout the placenta, coexisting with a normal living fetus. Case report. Br J Obstet Gynaecol.

[13] Hsieh CC, Hsieh TT, Hsueh C, Kuo DM, Lo LM, Hung TH (1999). Delivery of a severely anaemic fetus after partial molar pregnancy: clinical and ultrasonographic findings. Hum Reprod.

[14] Baxi LV, Mansukhani M, Thaker HM, Parravicini E (2014). Complete hydatidiform mole and live fetus in a singleton pregnancy with confined placental mosaicism and fetomaternal hemorrhage: a case report. J Reprod Med.

[15] Kawasaki K, Kondoh E, Minamiguchi S, Matsuda F, Higasa K, Fujita K (2016). Live-born diploid fetus complicated with partial molar pregnancy presenting with pre-eclampsia, maternal anemia, and seemingly huge placenta: a rare case of confined placental mosaicism and literature review. J Obstet Gynaecol Res.

[16] Sun CJ, Zhao YP, Yu S, Fan L, Wu QQ, Li GH (2012). Twin pregnancy and partial hydatidiform mole following in vitro fertilization and embryos transfer: a novel case of placental mosaicism. Chin Med J.

[17] Gajewska M, Zygula A, Wielgos M, Szewczyk G (2020). Twin pregnancy with a partial hydatidiform mole and a coexistent live fetus. Diagnostic and therapeutic dilemmas. A case report and the review of literature. Ginekol Pol.

[18] Jones WB, Lauersen NH (1975). Hydatidiform mole with coexistent fetus. Am J Obstet Gynecol.

[19] Fishman DA, Padilla LA, Keh P, Cohen L, Frederiksen M, Lurain JR (1998). Management of twin pregnancies consisting of a complete hydatidiform mole and normal fetus. Obstet Gynecol.

[20] Tasci Y, Dilbaz S, Secilmis O, Dilbaz B, Ozfuttu A, Haberal A (2005). Routine histopathologic analysis of product of conception following first-trimester spontaneous miscarriages. J Obstet Gynaecol Res.

[21] Zilberman Sharon N, Maymon R, Melcer Y, Jauniaux E (2020). Obstetric outcomes of twin pregnancies presenting with a complete hydatidiform mole and coexistent normal fetus: a systematic review and meta-analysis. BJOG..

[22] Buck Louis GM, Grewal J, Albert PS, Sciscione A, Wing DA, Grobman WA (2015). Racial/ethnic standards for fetal growth: the NICHD fetal growth studies. Am J Obstet Gynecol.

[23] Eggenhuizen GM, Go A, Koster MPH, Baart EB, Galjaard RJ. Confined placental mosaicism and the association with pregnancy outcome and fetal growth: a review of the literature. Hum Reprod Update. 2021. 10.1093/humupd/dmab009. 10.1093/humupd/dmab009 PMC8382909 33984128

[24] Makrydimas G, Sebire NJ, Thornton SE, Zagorianakou N, Lolis D, Fisher RA (2002). Complete hydatidiform mole and normal live birth: a novel case of confined placental mosaicism: case report. Hum Reprod.

[25] Gupta K, Venkatesan B, Kumaresan M, Chandra T (2015). Early detection by ultrasound of partial Hydatidiform mole with a coexistent live fetus. WMJ..

[26] Bifulco C, Johnson C, Hao L, Kermalli H, Bell S, Hui P (2008). Genotypic analysis of hydatidiform mole: an accurate and practical method of diagnosis. Am J Surg Pathol.

[27] Nayeri UA, West AB, Grossetta Nardini HK, Copel JA, Sfakianaki AK (2013). Systematic review of sonographic findings of placental mesenchymal dysplasia and subsequent pregnancy outcome. Ultrasound Obstet Gynecol.

[28] Busca A, Parra-Herran C. Partial hydatidiform mole. PathologyOutlines.com website. https://www.pathologyoutlines.com/topic/placentaincompletemole.html. Accessed 9 Mar 2021.

[29] Braga A, Obeica B, Werner H (2017). A twin pregnancy with a hydatidiform mole and a coexisting live fetus: prenatal diagnosis, treatment, and follow-up. J Ultrason.

[30] Ronnett BM (2018). Hydatidiform moles: ancillary techniques to refine diagnosis. Arch Pathol Lab Med.

[31] Fukunaga M, Katabuchi H, Nagasaka T, Mikami Y, Minamiguchi S, Lage JM (2005). Interobserver and intraobserver variability in the diagnosis of hydatidiform mole. Am J Surg Pathol.

[32] Colpaert RM, Ramseyer AM, Luu T, Quick CM, Frye LT, Magann EF (2019). Diagnosis and Management of Placental Mesenchymal Disease. A review of the literature. Obstet Gynecol Surv.

[33] Han LM, Grenert JP, Wiita AP, Quinn M, Fujimoto VY, Rabban JT (2020). Prevalence of partial Hydatidiform mole in products of conception from gestations with fetal Triploidy merits reflex genotype testing independent of the morphologic appearance of the chorionic villi. Am J Surg Pathol.

[34] Buza N, Hui P (2021). Genotyping diagnosis of gestational trophoblastic disease: frontiers in precision medicine. Mod Pathol.

[35] Xing D, Adams E, Huang J, Ronnett BM (2021). Refined diagnosis of hydatidiform moles with p57 immunohistochemistry and molecular genotyping: updated analysis of a prospective series of 2217 cases. Mod Pathol.

[36] Xie Y, Pei X, Dong Y, Wu H, Wu J, Shi H (2016). Single nucleotide polymorphism-based microarray analysis for the diagnosis of hydatidiform moles. Mol Med Rep.

[37] DeScipio C, Haley L, Beierl K, Pandit AP, Murphy KM, Ronnett BM (2011). Diandric triploid hydatidiform mole with loss of maternal chromosome 11. Am J Surg Pathol.

[38] Fisher RA, Nucci MR, Thaker HM, Weremowicz S, Genest DR, Castrillon DH (2004). Complete hydatidiform mole retaining a chromosome 11 of maternal origin: molecular genetic analysis of a case. Mod Pathol.

[39] Monk D (2015). Genomic imprinting in the human placenta. Am J Obstet Gynecol.

[40] Demond H, Anvar Z, Jahromi BN, Sparago A, Verma A, Davari M (2019). A KHDC3L mutation resulting in recurrent hydatidiform mole causes genome-wide DNA methylation loss in oocytes and persistent imprinting defects post-fertilisation. Genome Med.

[41] Zhang RQ, Zhang JR, Li SD (2019). Termination of a partial hydatidiform mole and coexisting fetus: a case report. World J Clin Cases.

[42] Zeng C, Chen Y, Zhao L, Wan B (2019). Partial Hydatidiform mole and coexistent live fetus: a case report and review of the literature. Open Med (Wars).

[43] De Franciscis P, Schiattarella A, Labriola D, Tammaro C, Messalli EM, La Mantia E (2019). A partial molar pregnancy associated with a fetus with intrauterine growth restriction delivered at 31 weeks: a case report. J Med Case Rep.

[44] Loza AJ, Fang YMV (2019). Complete molar pregnancy coexisting with a normal fetus in the third trimester. Am J Obstet Gynecol.

[45] Gabra MG, Gonzalez MG, Bullock HN, Hill MG (2020). Cell-free DNA as an addition to ultrasound for screening of a complete Hydatidiform mole and coexisting Normal fetus pregnancy: a case report. AJP Rep.

[46] Wang Y, Qian H, Wang J (2015). Medical termination of a partial hydatidiform mole and coexisting fetus during the second trimester: a case report. Oncol Lett.

[47] Niemann I, Sunde L, Petersen LK (2007). Evaluation of the risk of persistent trophoblastic disease after twin pregnancy with diploid hydatidiform mole and coexisting normal fetus. Am J Obstet Gynecol.

[48] Ray A, Kumari S (2020). A case report of twin pregnancy with Hydatidiform mole and co-existing live fetus. Saudi J Med Med Sci.

[49] Gestational Trophoblastic Neoplasia, Version 2.2021, NCCN Clinical Practice Guidelines in Oncology. https://www.nccn.org/guidelines/guidelines-detail?category=1&id=1489. 10.6004/jnccn.2019.0053 31693991

